# Determination of Mirtazapine and Desmethyl Mirtazapine in Human Plasma by a New Validated HPLC Ultraviolet Method with a Simple and Reliable Extraction Method: Application to Therapeutic Drug Monitoring Study by 62 Real Patient Plasma

**DOI:** 10.22037/ijpr.2019.14599.12519

**Published:** 2020

**Authors:** Emrah Dural, Nilay Sedes Baskak, Hatice Özcan, Yağmur Kır, Bora Başkak, Halil Sinan Süzen

**Affiliations:** a *Department of Pharmaceutical Toxicology, Faculty of Pharmacy, Sivas Cumhuriyet University, Sivas, Turkey. *; b *Department of Psychiatry, Yenimahalle Research and Training Hospital, Ankara Yıldırım Beyazıt University, Ankara, Turkey. *; c *Department of Forensic Toxicology, Institute of Forensic Sciences, Ankara University, Ankara, Turkey. *; d *Department of Psychiatry, Faculty of Medicine, Ankara University, Ankara, Turkey. *; e *Department of Pharmaceutical Toxicology, Faculty of Pharmacy, Ankara University, Ankara, Turkey.*

**Keywords:** Mirtazapine, N-desmethylmirtazapine, Plasma, HPLC-UV, Therapeutic drug monitoring, Validation

## Abstract

Determination of mirtazapine (MRP) during psychopharmacotherapy in biological fluids is essential to achieve successful therapy, to avoid toxicity related to drug interactions, genetic variability, and poor compliance. A new, rapid, and sensitive high-performance liquid chromatography method has been developed in human plasma for the determination of MRP and *N*-desmethylmirtazapine (NDM) that is an active metabolite.

The separation was achieved on a reverse-phase C18 250 x 4.6 mm i.d., ODS-3 column using programmed gradient elution at 40 °C. 20 mM potassium phosphate buffer (pH 3.9), acetonitrile, and triethylamine (75.0:24.9:0.1, v/v/v) were used as mobile phase A. Mobile phase B consisted of absolute acetonitrile. Clozapine was used as an internal standard. The method showed linearity with good determination coefficients (r^2^≥0.9981) for each analyte. Intra-day and interday assay precisions (RSD%) were found less than 3.4 and 2.9 for MRP and NDM, respectively. The intra-day and interday accuracy (RE%) of the method were calculated between (-2.8) and 5.5. A new extraction method was used in the study and an excellent recovery (average) values for MRP and NDM (94.4%, 106.6%, respectively) was obtained. The method was specific and sensitive as the limit of detection (LOD) were 0.17 for MRP and 0.15 ng/mL for NDM.

This method was applied properly to plasma samples taken from patients receiving MRI (n = 62) treated with 15-30 mg / day. The obtained and statistically evaluated plasma MRP and NDM levels which were 28.6 ± 13.8 and 12.3 ± 6.5 (mean ± SD). The described procedure is relatively simple, precise, and applicable for routine therapeutic drug monitoring especially in psychiatry clinics and toxicology reference laboratories.

## Introduction

**Figure 1:**
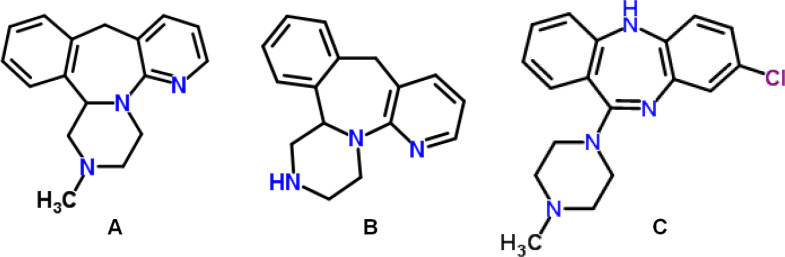
Chemical structure of MRP (a), of NDM (b), and CLZ (c) as the ISTD

MRP, a member of tetracyclic piperazinoazepine [1,2,3,4,10,14b-hexahydro-2-methyl-pyrazino[2,1-a]pyrido[2,3-c]benzazepine] (Figure 1.a), is a well-tolerated tetracyclic antidepressant that is used in the treatment of major depressive disorder (1, 2). Since MRP combines two synergistic mechanisms of action that enhance both noradrenergic and specific serotonergic neurotransmissions - referred to as NaSSA (3), the drug has a unique profile of action that is unrelated to a known psychotropic drug class (4). Particularly, pre-synaptic α2-adrenergic receptors, postsynaptic serotonin type 2 (5-HT2), and type 3 (5-HT3) receptors are blocked by MRP (5). Since MRP also has high-affinity to receptors of histamine H1, it causes sedation, increased appetite, and weight gain (1, 6). 

Due to its superior efficacy in the short-term and continuous treatment of moderately and severely depressed hospitalized and out-patients, MRP is a commonly prescribed antidepressant used in major depressive disorder (4). In addition to this, MRP is also a choice of treatment in other psychiatric disorders including generalized anxiety disorder, obsessive-compulsive disorder, and post-traumatic stress disorder (6). Because of its antiemetic properties, MRP may promise to prevent post-chemotherapy nausea and vomiting (2).

MRP is quickly absorbed after either single or multiple oral administrations with a peak plasma concentration reached within 2 h. MRP is extensively metabolized in the liver by the CYP2D6, CYP3A4, and CYP1A2 isoforms (6), it does not exert a potent inhibitor or inducer effect on any of these enzymes (7). The bio-transformation of MRP includes *N*(2)-demethylation, 8-hydroxylation, *N*(2)-oxidation, and sulphate or glucuronic acid conjugation (1, 4). MRP has two main metabolic pathways that are demethylation and oxidation (6). NDM, called nordesmethylmirtazapine, (Figure 1.b) is a pharmacologically active major metabolite, that contributes to approximately 15% of the total pharmacodynamic profile (6, 8). 

MRP is a basic, lipophilic drug with a pKa value 7.1 (6). For oral administration, it is available in the form of tablets or solutions (Remeron®), containing 15, 30, and 45 mg of MRP. 15 mg/day that is the initial dose of MRP, can be increased up to 80 mg/day (1, 2, 9, 10) based on the treatment response. Corresponding to these daily doses, typical plasma levels are between the 30-80 ng/mL (2). Diet has little effect on the rate of absorption and absolute bioavailability is approximately 50% after a single oral dose in humans (6, 9). In a non-specific and reversible way, MRP binds to plasma proteins with the ratio of 85%, and peak plasma concentration is achieved within 2 h of dosing (5, 9). MRP elimination half-life range is approximately between 20 and 40 h, those 26 and 37 h for male and females, respectively. Since elderly and females patients show higher plasma concentration than young adults and males, it is considered that MRP pharmacokinetics depends on age and gender (9). MRP has a long plasma half-life, and is usually taken once daily, immediately before sleep (2). Significant differences in the pharmacokinetics of MRP among individuals indicate that therapeutic drug monitoring is very important in terms of individual variability in treatment response as well as compliance. 

Some studies suggest that Suicide attempters may be overrepresented among major depressive disorder patients under MRP treatment (11–14), which may be associated with serum concentration of the drug. The drug concentration is also important in terms of toxicity. Contradictory findings were reported on the effects of MRP overdose. There are case studies that associate rhabdomyolysis with high concentrations of MRP in body fluids suggesting that drug toxicity may end up with a “serotonin syndrome”. On the other hand, some studies report that MRP toxicity is a relatively benign clinical condition characterized by slight CNS depression, tachycardia, and slight hypertension. Some studies have suggested that MRP is relatively non-toxic (15).

As a result, it seems essential to use a specific and rapid method for the determination of MRP and NDM not only for therapeutic drug monitoring, but also to evaluate side effects and toxicity. Several methods have been described in the literature for the determination of MRP and NDM in biological fluids. These are spectrophotometric (2, 4), capillary electrophoresis (4), gas chromatography (14, 16, 17), and high-performance liquid chromatography (HPLC) with ultraviolet detector, (4, 5, 18–20) diode array detector, (8, 21–24), fluorescent detector (3, 25, 26), and tandem mass spectrometry (27). However, long duration of analysis, sample preparation protocols, and sample quantities required for these analyses limit their use. At the same time, low sensitivity, precision, and accuracy values may indicate that they are unsuitable within the bounds for intraday and inter-day repeatable analysis. 

The greatest methodological disadvantage of the protocols previously described in the literature is the extensive labour-intensive liquid-liquid extraction during sample preparation (3, 18). Some studies used plasma samples in high amounts that is difficult to be collected in routine clinical practice (3, 5, 21). As chromatographically the long analysis time of the Frahnert *et al*. (2003) study can adversely affect the formation of rapid analysis results, especially in the case of high-dose poisons, which are toxicological precautions (18). Lavasani *et al.* (2014) (6) and Duvernoil *et al.* (2003) (22) methods have inadequate detection and quantitation limits, it may have resulted in insufficient and incorrect results, especially in blood samples with MRP and NDM at low concentrations. The high RE % and RSD% values of some studies (5, 18) in the daily and inter-day accuracy and precision values of the studies may adversely affect the achievement of the correct analysis result. The fact that the linear range of some methods is incompatible with the expected therapeutic values in patient plasma also may limit the applicability of the methods (5). Consequently, the validation test results have high standard deviations which may also have a negative effect on the results.

This paper describes a unique and reliable RP - HPLC with ultraviolet detection (UV) method for the simultaneous measurement of MRP and NDM in plasma involving an internal standard, clozapine. The proposed method presents several important advantages, such as relatively basic sample preparation procedure, rapidity, selectivity, linearity in the therapeutic range and repeatability. It is also particularly adapted for the management of poisoning cases leading to concentrations generally greater than the therapeutic range. Rapid chromatographic separation allows a run time below 12 min. The limits of quantitation were below 0.6 ng/mL. After the method was optimized, it was validated by linearity, precision, accuracy, and robustness in accordance with the ICH guidelines (28) In our study, plasma MRP and NDM levels of patients (n = 5), who were under the depression treatment with 15 – 30 mg/day MRP (Remeron®) orally, were also successfully monitored by our developed and validated method.

## Experimental

*Chemicals and reagents*

Pure reference samples of MRP and NDM were obtained from Cerilliant Texas, USA. The pure reference sample of clozapine (CLZ) used as the internal standard (ISTD) (Figure 1.c) was purchased from Sigma-Aldrich (Missouri, USA). HPLC grade acetonitrile and methanol were obtained from Sigma-Aldrich (Steinheim, Germany). Hexane, isoamyl alcohol, triethyl amine, potassium dihydrogen phosphate, sodium hydroxide, orthophosphoric, and hydrochloric acid which are analytical grade were purchased from Merck (Darmstadt, Germany). Membrane filters pore size of 0.45 µm used as filtration of mobile phases obtained from Millipore (Massachusetts, USA). Ultrapure water was made by Elga Purelab (United Kingdom) ultrapure water system. Freshly human plasma used in the method development and validation procedures was provided from the Blood Transfusion Center of Medical School of Sivas Cumhuriyet University. 

*Instrumentation*

The separation and quantification were performed by Hewlett-Packard (HP) Agilent 1100 series (California, USA) high-performance liquid chromatography (HPLC) system which consist of a degasser (G1322A, Degasser), a gradient pump (G1311A, QuadPomp), a Rheodyne 7725i manual injector with 100 µL sample loop, a column oven (G1316A, Colcom), and an ultraviolet detector (G1314A, VWD). Analytical separation was performed by a C_18_ (250 x 4.6 mm, 5 µm particle size) GL Sciences (Tokyo, Japan) analytical column at 40 °C. The mobile phase A consisted of phosphate buffer (20 mM), acetonitrile, triethylamine (75:24:1, v/v/v) pH 4.3 and the mobile phase B consisted of absolute acetonitrile. Flow rate was 1.2 mL/min. The mobile phases were filtered through a 0.45-µL filter (Sartorius, Goettin, Germany) after pH adjustment with 1 M orthophosphoric acid. Then, it was degassed for 30 mins in an ultrasonic bath. The gradient mobile phase flow program was as follows: Initially, mobile phase A flow ratio is 60% during the 4.5 min. After 4.5 min, mobile phase B increasing linearly from 40% to 80% over 10.5 min, followed by isocratic elution as initial mobile phase condition over 5 min. The time elapsed between the two analyzes was 20 min. All compounds were chromatographed at 290 nm. The unknown concentrations of MRP and NDM were quantified using linear regression of response (drug/ISTD peak area) versus MRP or NDM concentrations. System control and integrated data were recorded using the Chemstation® computer software version of 08.3 (California, USA). 

*Standard solutions*

Stock solutions of MRP, NDM, and CLZ (1 mg/mL) were prepared in methanol and stored at -20 °C until use. They were observed that stable for at least 3 months. The working solutions of MRP and NDM were prepared weekly from the main stock solution with methanol as 0.5, 1, 1.5, 2, 3, 4, 5, 6 7.5, 12.5 µg/mL concentrations. MRP and NDM dilutions were freshly prepared into drug-free human plasma to provide concentrations of 10, 20, 30, 40, 60, 80, 100, 120, 150, and 250 ng/mL of both compounds. The ISTD main stock solution was diluted weekly with methanol to yield a 10 μg/mL working solution. Likewise, plasma quality control (QC) standards spiked with 60, 100, 150 ng/mL of both MRP and NDM were prepared to measure the repeatability values (accuracy and precision) of the method. Also the same protocol was used in preparation of limit of detection (LOD) and quantitation (LOQ) and robustness and recovery test samples. 

*Sample preparation*

10 µL CLZ (20 µg/mL) as an ISTD and then 200 µL NaOH (0.1 N) for the alkalization of the matrix were added to the 0.5 mL plasma for preparation of validation or patient samples. In addition, while preparation for QC standards, except for the preparation of patient plasma, 10 µL of the working solutions of MRP and NDM were added. The mixture was extracted with 5 mL hexane, isoamyl alcohol mixture (95:5, v/v) by the rotative shaking during 10 min and then centrifuged to 3500 rpm at 10 min. After centrifugation, the upper organic phase was separated to test tube containing 0.2 mL, 0.1 N HCl. The mixture was shaken with the rotative shaker during 10 min and after then centrifuged at 3500 rpm over 5 min. The upper organic phase was discarded and the 100 µL of 200 µL remaining acidic aqueous phase was loaded to the liquid chromatograph by manual injection system as 100 µL.

*Method validation*

The developed analytical method was validated to demonstrate the specificity and selectivity, linearity, accuracy and precision, limit of detection (LOD) and limit of quantification (LOQ), recovery and robustness. Intra- and inter-day validation protocol was applied considering reproducibility of method and instrument to obtain accurate and precise measurements in agreement with Conference on Harmonization guidelines (28). 

*Specificity and selectivity*

**Figure 2a:**
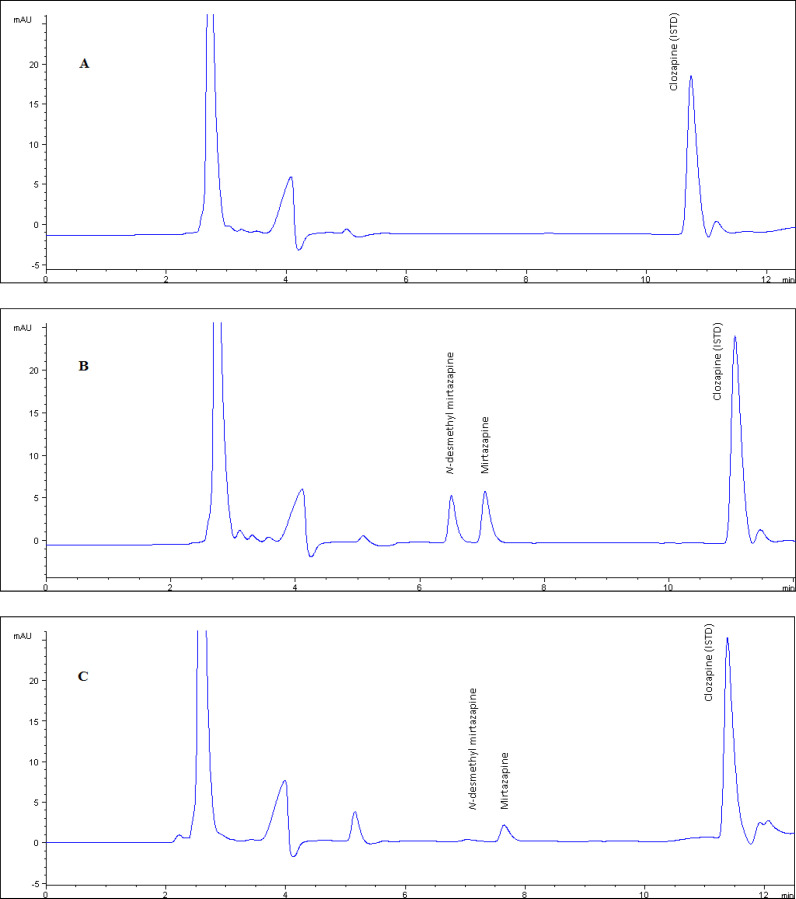
The chromatogram of blank plasma used in validation tests. Figure 2 b. The chromatogram of plasma example that contained NDM and MRP as 250 ng/mL concentration which is prepared by standard addition method used as quality control sample. Figure 2.c. The chromatogram of real plasma sample belongs to patient who was using 15 mg/day MRP

The method showed excellent chromato-graphic specificity without endogenous inter-ference at the retention times of NDM, MRP, and CLZ (6.7, 7.3, 11.3 min, respectively) in plasma. Representative chromatograms of blank (Figure 2.a), spiked (Figure 2.b) and patient samples (Figure 2.c) illustrate the high resolution in 12 min as the short separation time. The ultraviolet detection was set to 290 nm displaying for optimum sensitivity. 

*Linearity*

After chromatographic conditions were established, matrix-based calibration curves for MRP and NDM were plotted concentrations over the range 10 - 250 ng/mL versus peak-area ratios to the ISTD (CLZ). The calibration points (n = 10), which were 10, 20, 30, 40, 60, 80, 100, 120, 150, and 250 ng/mL, composed 3 individual replicates that were prepared by standard addition method in plasma and injected to HPLC. 

*Accuracy and precision*

The accuracy, defined as the relative error (bias %) was calculated as the percentage difference between the added and found MRP and NDM quantity by 5 separate replicates both intraday and inter-day. The precision, defined as relative standard deviation (RSD), was calculated by five separate replicates of MRP and NDM both intraday and inter-day. Five replicate spiked samples were assayed intraday and inter-day at the three different concentrations (40, 80 and 150 ng/mL) for all analytes. 

*Sensitivity*

The limit of detection (LOD) and limit of quantification (LOQ) were calculated according to the ICH recommendations (28) based on standard deviation of the response and the slope of the calibration graph. 

LOD= 3.3σ/S; LOQ= 10σ/S (σ: The standard deviation of the response; S: The slope of the calibration curve). The concentration of 10 ng/mL as the lowest calibration points was used in sensitivity tests of MRP and NDM. 

*Recovery*

The recovery of extraction procedures from human plasma was determined by comparing pre-extraction spikes with the post-extraction spiked ISTD. Five individual replicates of spiked samples at low, middle, and high concentrations (60, 150 and 250 ng/mL, respectively) of MRP and NDM were prepared with and without ISTD. Extraction recoveries were determined by comparison of extracted samples of MRP and NDM, to those from unextracted and directly injected standards, spiked with same amounts. Extraction procedure was carried out as described previously. 

*Robustness*

The response of the method to changes in ultraviolet wavelength (± 3 nm), mobile phase flow rate (± 0.1 mL / min), mobile phase organic solvent content (± 5%) and column temperature (± 4 °C) was observed. In addition, the chromatographic separation effect of changes in analyst, column, source of chemicals, or solvent wastested. 

*Stability*

The stability of QC plasma samples (40, 80 and 150 ng/mL) and analytes in stock solutions under several conditions was assessed. Room temperature stability of stock solutions was evaluated in 1, 2, 3 and 4 weeks. The stability test of freeze-thaw was executed by three QC samples after operating five repeated freeze-thaw period. The stability test of long-term was carried out for 1, 2 and 3 months using QC samples kept at -20 °C. 

*Collection of plasma samples*

4 mL of whole blood samples were taken from the patients with depression orally treated with MRP who have steady-state concentration in plasma. After it was centrifuged at 3500 rpm for 5 min, the obtained individual 2 mL plasma samples were stored in -80 °C until being analyzed. The study protocol was approved by Ankara University, Medical School Clinical Research Ethics Committee with 05-189-13 decision number on 7 March 2013 and was conducted in accordance with the Declaration of Helsinki and its subsequent revisions. Informed consent was obtained from all volunteers prior to inclusion in the research. The blood samples were obtained from 63 patients who were under the depression treatment at the Ankara University Medical School Department of Adult Psychiatry. The blood samples were drawn 12 h after the last drug administration. The blood was stored in glass tubes containing Na_2_EDTA, then centrifuged at 3500 rpm for 10 min; the supernatant (plasma) was transferred to polypropylene tubes and stored frozen at - 20 °C until analysis. Plasma MRP and NDM levels in the patients were measured in less than three months. 

## Results and Discussion

*Selection of ISTD*

Carbamazepine, fluphenazine, opipramol, imipramine, chlorpromazine, sildenafil, and CLZ were tested in the selection of the ISTD. Imipramine, fluphenazine and opipramol did not demonstrate the acceptable ultraviolet intensity. In addition to that carbamazepine and sildenafil did not show appropriate retention times and their peak shapes were not enough sharpened. Because of the obtained prescise and reproducible data from the satellite study by CLZ, it was selected as the ISTD. Also, CLZ showed so nice intensity results. In addition to its recovery values on extraction were both acceptable and reproducible. 

*Linearity*

**Figure 3:**
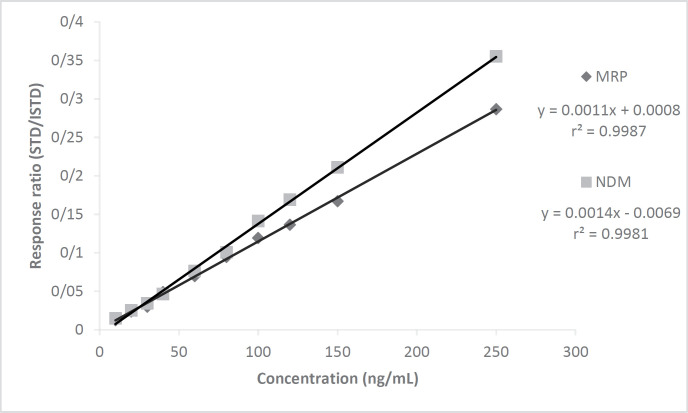
The calibration graphs for MRP and NDM prepared in plasma by standard addition method

Calibration curves of MRP and NDM were drawn at 10 points (n = 3) between 10 - 250 ng/mL concentration versus the area of CLZ as ISTD by the standard addition method showing excellent correlation with r^2^ = 0.9981 and 0.9987, respectively (Figure 3). The linearity study was designed to cover sub therapeutic, therapeutic, and toxic drug levels of the drug. The correlation values obtained at the individual 10 points was quite good.

*Accuracy and precision*

The data obtained from the accuracy and precision tests (Table 1), performed in intraday and inter-day with quality control standards established in the blank plasma samples by standard addition method showed low RSD ≤3.30 and RE% value between -2.82 and 5.50. 

*Sensitivity*

The results of LOD and LOQ values, which were obtained by the measurement of individual 10 quality control (QC) samples, (Table 2) demonstrated that developed method has very low sensitivity values. In addition, the system suitability parameters are given in Table 2. 

*Recovery*

Peak area ratios were compared and recoveries were calculated as between 91.86% and 108.79% for each analyte tabulated in Table 2. Recovery values indicate that the extraction procedure is well suited for routine analysis.

*3.6. Robustness*

No significant changes in the analytical signals were observed upon changing ultraviolet wavelength value (±3 nm), mobile phase flow rate (± 0.1 mL/min), mobile phase organic solvent ingredient (± 5%), column temperature (± 4 °C). However, changes in analysts, columns, sources of chemicals and/or solvents did not lead to significant changes in chromatographic signals, too. Robustness experiments demonstrated that the method created data of acceptable precision and accuracy. 

The validation test results and chromatographic conditions of studies investigating plasma MRP levels by HPLC in the literature are summarized in Table 3.

According to the HPLC methods in the literature up to now, the lowest quantitation limit value was found in the method of Lavasani *et al*. (2014) (6) with 1 ng/mL for MRP and 2 ng/mL for NDM. However, the method we proposed is the lowest analytical method in the literature with quantitation limits of 0.52 ng/mL for MRP and 0.46 ng/mL for NDM. Our method will prevent the “false negative” and “not detected” trouble. Our method of precision values (RSD%) that are below the 3.30% and 2.89% (in intraday and in inter-day respectively) are the lowest RSD values for MRP and NDM measurements in the literature so far. Intraday and inter-day accuracy values (RE%) are between (-2.82) and 3.45; (-0.62) and 5.50 for MRP and NDM in our study. The recovery values of the proposed method are 94.40% for MRP and 106.98% for NDM at concentrations of low (20 ng / mL), medium (60 ng / mL) and high (100 ng / mL) concentrations. Duvernoil *et al*. (2003) (22) shows higher recovery values with 102% for MRP in the literature. Compatible with literature, in our study, a buffer with low pH (3.9), 0.1% triethyl amine and acetonitrile were applied the analytical column with gradiently, and the analysis was performed in less than 12 min. The method with the shortest duration of analysis in the literature is Lavasani *et al.* (2014) (6) is a 5-minute method, but there is a 2 mL mobile phase flow per minute as a gradiently in their method. The method we propose has been validated to be used in the management of high-dose linked poisoning cases with MRP. The linear range of the method is between 10 ng / mL and 250 ng / mL for MRP and NDM. A 500 μL plasma sample was used during the sample preparation phase of our study. The lowest plasma volume which used in analyses in the literature belong to Lavasani *et al.* (2014) (6) which is 150 µL. The volume of plasma is 1000 μL frequently used in methods developed for the analysis of MRP and its metabolites. However, considering the difficulty in obtaining high amounts of the blood samples from patients, analysis methods below 1 mL of plasma volume are preferred. The study of Romingueres *et al.* (2002) (5) has the shortest retention time for the analysis of MRP and NDM. In our proposed method, the retention times of analytes MRP, NDM and CLZ as an ISTD were 6.5, 7.1 and 11.1 min and the total analysis time is below 12 min. That time is sufficient for therapeutic drug monitoring and toxicological analysis of MRP. This time is also short enough for the monitoring of the therapeutic drug level of MRP and the analysis of toxicological purpose of this drug in plasma (Table 3). 

*Stability*

Neither significant decrease nor degradation were observed in the concentration of MRP and NDM in three different conditions (at room temperature for 4 weeks, -20 °C for 3 months and after five repeated freeze-thaw period). The relative standard deviation in all samples was less than 4%. 

*Measurement of patient plasma samples treated with MRP*

MRP and NDM levels in blood samples taken from patients who were receiving orally 15 and 30 mg/day MRP (Remeron®) for depression treatment were monitored by developed and validated HPLC method. The analyses were performed by 0.5 mL plasma samples of the patients with plasma steady-state concentration of MRP in their plasma. None of these samples showed any problem for the quantification of the analytes, additionally, peak purity showed that no analytical interference was encountered from endogenous substances. The obtained results are given in Table-4, 5 and 6.

Patient plasma MRP and NDM levels were successfully analyzed, that a sample chromatogram was given in Figure 2.c, by the developed method. Although the blood samples were taken from the patients (n = 62) treated with 15-30 mg/day orally administration (Remeron®), MRP and its metabolite NDM levels 28.6 ± 13.8 and 12.3 ± 6.5 respectively) widely varied between the individuals. Metabolic differences between individuals are thought to play an important role in the emergence of this difference. In particular, the decrease in the amount of MRP absorbed by the first pass effect negatively affects the bioavailability. This will cause the pharmacological activity to be reduced. 

There was no significant difference between MRP and main metabolite concentrations in plasma samples of the male and female patients (*p* > 0.05). Moreover, there was no significant difference between the age of the patients and plasma drug levels (*p *> 0.05). On the other hand, the results obtained indicate that the obtained MRP is represented by metabolite NDM at an average rate of 42.08 % in plasma. However, significant inter-individual differences in NDM levels (12.3 ± 6.5, mean ± SD) suggest that phase I enzyme may also be associated with inter individual variation in the efficacy of CYP3A4. 

**Table 1:** Confidence parameters of validated method; intra-day and inter-day precision and accuracy for determination of MRP and NDM in human plasma samples

	Conc. (ng/mL)	**Intra-day**				**Inter-day**			
		*No.* *Obs.*	Estimated conc. ±SD (ng/mL)	Precision (RSD%)	Accuracy (RE%)	*No.* *Obs.*	Estimated conc. ±SD (ng/mL)	Precision (RSD%)	Accuracy (RE%)
MRP	40	5	43.62 ± 0.74	3.30	-0.57	5	39.75 ± 5.51	1.68	-0.62
	80	5	95.52 ± 1.73	2.56	0.69	5	81.92 ± 6.28	1.76	2.40
	150	5	147.67 ± 73.72	3.35	0.23	5	158.25 ± 8.01	2.01	5.50
NDM	40	5	41.38 ± 0.28	1.07	3.45	5	40.80 ± 0.27	2.40	1.99
	80	5	80.44 ± 1.58	2.85	0.55	5	82.96 ± 1.14	2.23	3.69
150	5	145.77 ± 1.02	1.19	-2.82	5	151.75 ± 3.69	2.89	1.17

**Table 2:** System suitability parameters, sensitivity and recovery values of the method

**Analytes**	**Capacity factor (k')**	**Theoretical plate number** **(** ***N*** **)**	**Selectivity** **factor** **(α)**	**LOD** **(ng/mL)**	**LOQ** **(ng/mL)**	**Recovery (%)**		
						20 ng/mL	60 ng/mL	100 ng/mL
MRP	1.7	357.3	1.9	0.17	0.52	91.86	94.35	97.00
NDM	1.4	389.3	2.3	0.15	0.46	108.79	105.53	106.63
CLZ	3.2	452.0	-	-	-	-	-	-

**Table 3:** The validation parameters and chromatographic properties of MRP and NDM determination methods from plasma samples in literature

**Study**	**Analytes**	**LOQ (ng/mL)**	**Accuracy (RE%)**	**Precision (RSD%)**	**Recovery (%)**	**Retention times (min)**	**Linearity (ng/mL)**	**Mobile phase**	**Extraction type**	**Flow (mL/min)**	**Instrument - detector**	**Amount of used plasma (µL)**	**Total analysis time (min)**
Ptacek *et al.* (2003) [3]	MRP	1.5	(-1.2) – (6.5)	MRP, ≤4.9	-	-	2 - 151	ACN:30mM PB (2% TEA and SA) pH:4.0	LLE	1	HPLC-FL	1000	4
	NDM		-	-	-	-	-						
Romingueres *et al.* (2002) [5]	MRP	20	(-4.2) – 2.0	≤8.3	96.9	4.2	50 - 500	ACN: 6.24 mM PB, pH:6.4	LLE	0.8	HPLC-UV	1000	12
	NDM	20	(-9.9) – 1.1	≤6.1	102.9	2.6	50 - 500						
Lavasani et al (2014) [6]	MRP	1	(-9.98) - 18.3	≤12.9	86.9	3.5	1 - 500	ACN: 25mM PB, pH:3.0 (20:80, v/v)	LLE	2	HPLC-FL	150	5
	NDM	2	(-18.9) - 6.8	≤16.7	37.2	3.0	2 - 500						
Frahnert *et al.* (2003) [18]	MRP	-	-	≤7.7	97.9	16.6	5 - 300	ACN: 25mM PB pH:7.0 (40:60, v/v)	SPE	1	HPLC-UV	1000	-
	NDM	-	-	-	-	8.3	-						
Titier *et al.* (2003) [21]	MRP	25	(-5.5) – (-5.1)	≤11.5	80.5	5.8	25-500	ACN: 10mM PB pH 3.8	LLE	1	HPLC-PDA	1000	18
	NDM	25	(-5.5) – (-3.0)	≤12.0	64.0	5.4	25-500						
Duvernoil *et al.* (2003) [22]	MRP	25	(-0.6) – 5.3	≤4.74	102.0	6.5	25 - 1000	ACN: PB pH:3.8	LLE	1	HPLC-PDA	1000	18
	NDM	20	-	-	-	-	-						
Pongtanya *et al.* (2012) [23]	MRP	3	<15	<15	-	-	3 - 300	MeOH:water (0.00002% TFA)	LLE	1	HPLC-DAD	1000	-
	NDM	-	-	-	-	-	-						
Dallet *et al.* (2002) [24]	MRP	-	-	-	-	-	-	ACN: 25mM PB, pH: 4.8 (10mM TEA) (66:35, v/v)	-	1	HPLC-PDA	-	20
	NDM	-	-	-	-	-	-						
Our developed method	MRP	0.52	(-0.6) – 5.5	< 3.4	94.4	7.3	10 – 250	MP-A: ACN: 20 mM PB, triethylamine (75:24:1, v/v/v) pH 4.3 MP-B: ACN	LLE	1.2	HPLC-UV	500	12
	NDM	0.46	(-2.8) – 3.7	< 2.9	107.0	6.7	10 - 250						

ACN: Acetonitrile; PB: Phosphate buffer; LLE: Liquid-liquid extraction; SPE: Solid-phase extraction; HPLC: High performance liquid

chromatography; FL: Flourescent; PDA: Photo diod array; TEA: Triethylamine, TFA: Triflouroacetic acid, SA: Sodium azide, MP: mobile phase.

**Table 4.** Number of samples and patients which were examined for MRP and NDM

**Samples (n)**	**Patients (n Total; Women %)**	**Samples with not Detectable Parent Substance**
63	38 (60.3)	1

**Table 5:** Number of samples included in the analysis, demographic data of the patients evaluated, median daily MRP doses and median serum concentrations

**Samples (n)**	**Women (%)**	**Median age in yrs (range)**	**>65 yrs, n (%)**	**Median dose (minimum to maximum) in mg/day**	**Median concentration (10 th – 90 th percentile) in ng/mL**
63	60.3	37.5	2 (3.2)	30 (15 – 30)	25.3 (10.0 – 51.3)

**Table 6:** MRP and main metabolite serum concentrations (ng/mL; 10 th, and 90 th, percentiles) at different daily doses

**MRP**		
Dose	15 mg	30 mg
Number of samples	19	19
10%	15.6	13.1
Median	25.1	25.4
90%	38.6	54.6
NDM		
10%	6.0	4.0
Median	10.5	10.8
90%	18.6	20.7

## Conclusions

The HPLC method developed in this article is rapid, specific, and sensitive. The precision and accuracy test result of the method, which are RSD ≤3.30 and %RE value between -2.82 and 5.50 respectively, have very good results (28), therefore reliability of the developed method is very high. The excellent recovery values between 94.35% and 106.63% obtained will attract the use of the method. Because of the simplicity of the sample preparation, short analysis time (<12 min) and the high sensitivity of presented technique make particularly attractive for the quantification of MRP and its major metabolite in human plasma. We strongly recommended this validated method to be used in routine therapeutic drug analysis of MRP and also it can be adapted for monitoring of overdose/poisoning with this drug in suicide cases. The proposed method can be easily applied in routine therapeutic monitoring of MRP, besides TDM, the stated method can be also very useful for bioequivalence studies, pharmacovigilance, and pharmacokinetics studies. Because of the significant differences observed in plasma drug and main metabolite levels, the polymorphism rates of CYP2D6, CYP3A4, and CYP1A2 subtypes responsible for MRP metabolism are going to be determined with our planned clinical study.
